# Unilateral cervical carcinoma in a septate uterus with double cervix: A case report and literature review

**DOI:** 10.1016/j.gore.2026.102054

**Published:** 2026-02-27

**Authors:** Camilla Bandeira Soares, Nick M. Spirtos, Joao Jabbur Stern, Marcelle Almeida Souza, Felipe Jacyntho Laterça, Gustavo Guitmann

**Affiliations:** a Department of Gynecologic Oncology, Americas Medical City, Rio de Janeiro, Brazil; b University Cancer Center and the Women’s Cancer Center of Nevada, University of Nevada, Las Vegas School of Medicine, USA; c Radiology Department, University Hospitals, Cleveland, OH, USA; d Department of Gynecologic Oncology, Brazilian National Cancer Institute (INCa), Coordinator of Gynecologic Oncology, Americas Medical City, Rio de Janeiro, Brazil

**Keywords:** Uterine cervical neoplasms, Uterine duplication anomalies, Congenital abnormalities, Septate uterus, Uterine didelphys

## Abstract

**Background:**

The exact incidence of cervical cancer occurring in patients with Müllerian anomalies is unknown as both conditions are relatively rare and the literature is compromised principally of individual case reports or small series. Despite the rarity of this coexistence, the literature consistently highlights the significant diagnostic and therapeutic challenges it presents. This is particularly true when cervical duplication is present as the altered anatomy can hinder adequate cytologic sampling and delay the detection of malignant lesions.

**Case presentation:**

We report a case of unilateral invasive endocervical adenocarcinoma in a 43-year-old patient with complete septate uterus and longitudinal vaginal septum. The tumor was confined to the left cervix, staged as FIGO IB2 and underwent a type C1 radical hysterectomy with bilateral salpingo-oophorectomy and pelvic and *para*-aortic lymphadenectomy. Following the histologic confirmation of a well-differentiated adenocarcinoma with lymphovascular invasion and pelvic nodal metastasis the patient underwent chemoradiation and remains disease-free at five years.

**Conclusion:**

This case highlights how cervical duplication, particularly in the presence of a longitudinal vaginal septum may compromise routine screening and underscores the importance of careful anatomic assessment to ensure accurate diagnosis and individualized management in patients with uncommon Müllerian variants.

## Highlights

• Unilateral cervical carcinoma can occur in patients with cervical duplication.
• Cervical duplication may compromise routine cytologic screening.
• Altered anatomy poses significant diagnostic and therapeutic challenges.
• Individualized surgical planning is essential in Müllerian anomalies.
• Long-term disease control is achievable with multimodal treatment.

## Graphical abstract

**Figure:**
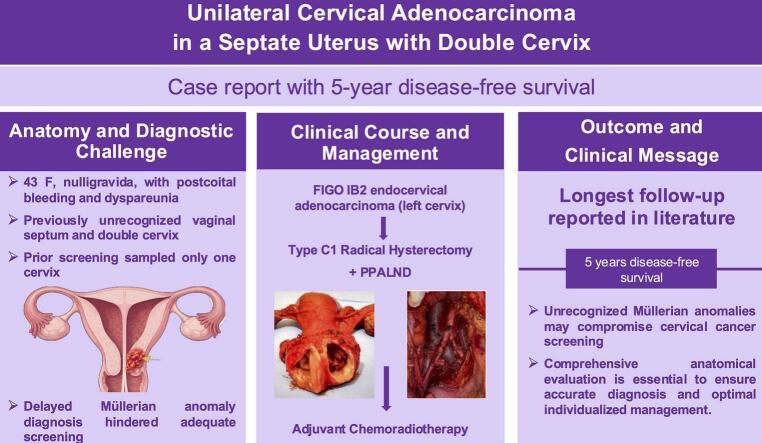

## 1 Introduction

Despite being largely detectable, if not preventable and in the early stages, cervical cancer remains one of the most common malignancies worldwide in middle and low-income countries due to the disparity, when compared to high -income countries, in the availability of regular cytologic screening. Its clinical presentation may be significantly altered in women with congenital Müllerian anomalies and in whom abnormal anatomy may hinder adequate cervical screening and lead to delayed diagnosis.

Müllerian duct anomalies arise from failures of fusion or resorption of the Müllerian ducts and may involve the uterus, cervix, vagina, and fallopian tubes. And affect approximately 1% of women. Uterine septation accounts for more than half of these malformations (Acién and Acién, 2016, American Fertility Society, 1988). Cervical duplication—historically associated almost exclusively with uterine didelphy, but has also been described in patients with incomplete uterine septation prompting revision of traditional embryologic theories (Acién and Acién, 2016). The 2013 ESHRE/ESGE classification reinforced this understanding by independently categorizing uterine, cervical, and vaginal anomalies (Grimbizis, 2013).

The coexistence of Müllerian anomalies and cervical cancer is rare and remains a diagnostic challenge. Altered anatomy may result in unilateral cytologic sampling, missed lesions, and errors in staging and suboptimal treatment. Most reports describe bilateral cervical involvement whereas, invasive unilateral tumors in the setting of cervical duplication are uncommon (Corbett et al., 1982, Gomez-Irizarry et al., 1996).

We present a case of unilateral endocervical adenocarcinoma in a patient with a uterine didelphys (complete uterine septation, double cervix, and longitudinal vaginal septum), followed by a review of similar reported cases and a discussion of the diagnostic and therapeutic implications of this unique anatomical configuration.

## 2 Case presentation

The patient was a 43-year-old nulligravid woman with a known uterine anomaly first suspected in November 2017 during transvaginal ultrasound, which demonstrated duplicated endometrial echo suggestive of uterine septum. A diagnostic hysteroscopy performed shortly thereafter described a tubular uterine cavity with a single right tubal ostium visualized, leading to suspicion of a unicornuate uterus and recommendation for pelvic MRI correlation. However, MRI was not performed at that time. The hysteroscopic evaluation was conducted through the right cervix, and cervical duplication as well as longitudinal vaginal septum were not recognized. A cervical cytology performed in December 2016 was negative for intraepithelial lesion or malignancy. Available documentation indicates that routine cervical screening had been performed sampling only one cervix, as duplication was unrecognized. No associated renal or urinary tract anomalies were identified on prior imaging.

**Fig. 1:**
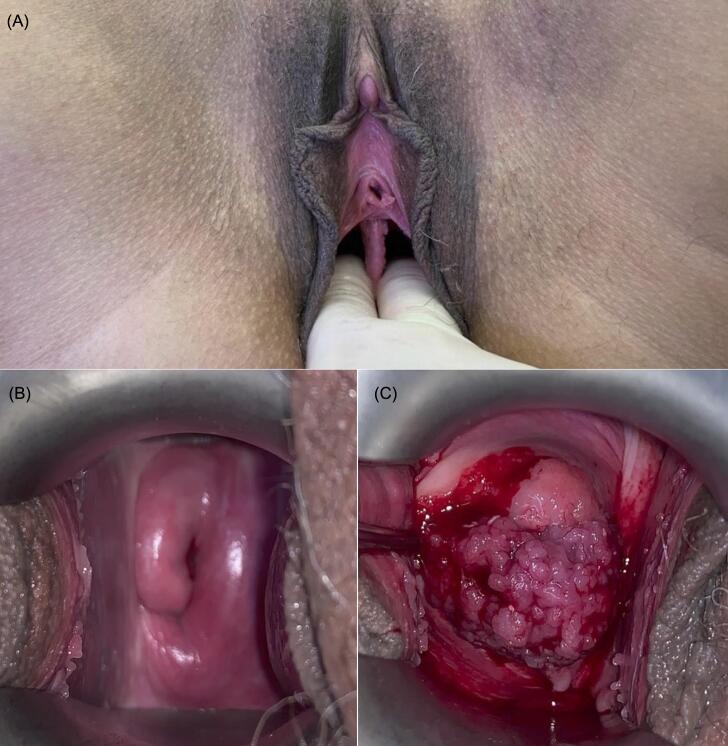
Presence of a longitudinal vaginal septum extending to the middle third of the canal (A). Right cervix without macroscopic abnormalities (B) and left cervix showing a friable, exophytic tumor (C) on initial gynecologic examination.

In March 2020, she presented with postcoital bleeding and dyspareunia and was evaluated at a gynecologic oncology service in Rio de Janeiro, Brazil. Pelvic examination revealed a longitudinal vaginal septum extending to the mid-vagina and two cervices, initially suggesting uterine didelphys. The right cervix appeared normal, while the left cervix had a friable, exophytic tumor measuring approximately 3.0 cm (Fig. 1), consistent with FIGO 2018 stage IB2 (cT1b2 N0 M0, AJCC 9th edition) (Committee, 2019, Olawaiye, 2021).

**Fig. 2:**
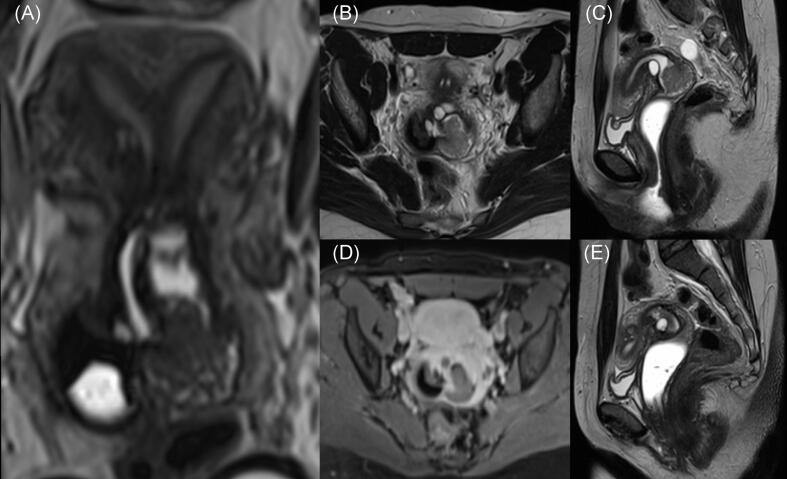
Magnetic resonance imaging of the abdomen. (A) 3D T2-weighted reconstructed image in the long axis showing a septate uterus with duplicated cervices and a longitudinal vaginal septum. The mass in the left cervix demonstrates intermediate T2 signal on axial (B) and sagittal (C) images and is hypovascular (D), consistent with cervical malignancy. (E) Sagittal T2-weighted image shows no suspicious lesions in the right cervix.

Pelvic MRI demonstrated a complete uterine septum dividing the endometrial cavity and an enhancing 3.0 × 2.6 × 2.7 cm mass arising from the left endocervical canal, with minimal extension into the proximal vagina with features typically associated with malignancy including poorly defined infiltrative borders, internal necrosis with cystic changes, and heterogenous signal intensity on T2 weighted images (Fig. 2).

PET-CT confirmed a hypermetabolic lesion confined to the left cervix without nodal or distant metastases. Prior to initiating treatment the patient was presented at a multidisciplinary conference and primary surgical management was recommended.

In June 2020, the patient underwent an examination under anesthesia which revealed a diffusely enlarged uterus, normal adnexa, double cervix, with the left involved by a cervical cancer approximately 3 cm in diameter as well as a longitudinal vaginal septum. Exploratory celiotomy confirmed these findings and further identified the absence of intra- or *retro*-preritoneal disease.

**Fig. 3:**
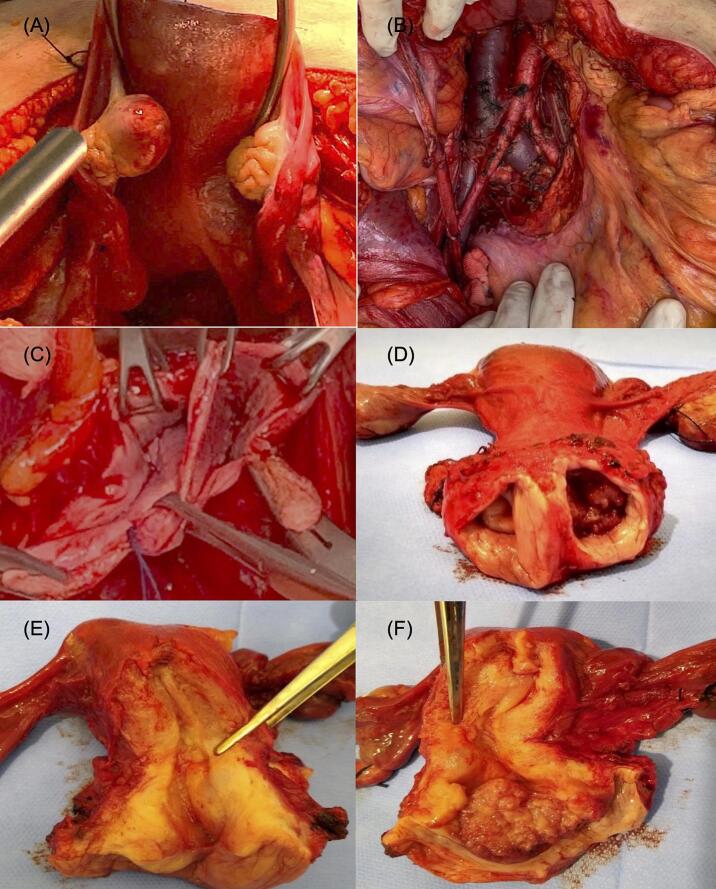
(A) Externally, the uterine fundus appears normal, and it is not possible to identify a cleft or separation between the endometrial cavities. (B) Retroperitoneum after lymphadenectomy. (C) Remaining longitudinal vaginal septum after radical hysterectomy and upper third colpectomy. (D) Uterine cervix with two ectocervices and two septated vaginal segments. (E) Right cervix without abnormalities. (F) Left ectocervix with a vegetative tumor involving its entire extent, without invasion of the uterine body.

The patient underwent a type C1 radical hysterectomy (Querleu–Morrow), bilateral salpingo-oophorectomy, and pelvic and *para*-aortic lymphadenectomy. The tumor measured 4.2 × 2.7 cm, with 10 mm of stromal invasion and lymphovascular space invasion. The right cervix, endometrium, adnexa, vagina, lymph nodes and all margins were noted to be without macroscopic evidence of disease. (Fig. 3).

Estimated blood loss was < 200 mL, and the postoperative recovery was uneventful, with discharge on postoperative day two. Bladder function returned on post-operative day 7 and the Foley catheter was discontinued.

Histopathology, however revealed a well-differentiated (G1) endocervical adenocarcinoma confined to the left cervix with metastatic disease was identified in one of six left pelvic lymph nodes, while all four right pelvic and ten *para*-aortic nodes were negative as were the surgical margins. Final staging was pT1b3 N1 M0 (FIGO IIIC1) (Committee, 2019, Olawaiye, 2021). Based on these findings the patient received pelvic radiotherapy (45 Gy in 25 fractions) with weekly cisplatin (40 mg/m^2^). At five-year follow-up, she remains asymptomatic with no evidence of disease.

This case represents a rare presentation of unilateral cervical adenocarcinoma in a patient with a complex Müllerian anomaly ultimately classified—per ASRM and ESHRE/ESGE criteria — as a complete septate uterus with cervical duplication and a longitudinal vaginal septum (Grimbizis, 2013).

## 3 Discussion

Müllerian duct anomalies result from developmental failures (uterine agenesis), incomplete fusion of the ducts (uterus, cervix and upper vagina) or resorption of vaginal septate, during embryogenesis. As a result, these anomalies present as a broad spectrum of uterine, cervical, and vaginal configurations. Septate uteri account for over half of these anomalies (Acién and Acién, 2016, American Fertility Society, 1988). Historically, the presence of duplicated cervices was interpreted as pathognomonic for uterus didelphys; however, modern frameworks have demonstrated that cervical duplication may also occur in septate and bicornuate uteri (Acién and Acién, 2016). The ESHRE/ESGE system (2013) improved diagnostic precision by independently classifying uterine, cervical, and vaginal components (Grimbizis, 2013). Based on this system, the present case corresponds to U2b/C2/V1, representing a complete septate uterus, cervical duplication, and a non-obstructive longitudinal vaginal septum.

Accurate classification is not merely descriptive; it has direct diagnostic implications. Cervical duplication may lead to unilateral cytologic sampling, incomplete colposcopic evaluation, and delayed recognition of dysplastic or malignant disease. Although cervical cancer is common worldwide, the coexistence of Müllerian anomalies and invasive cervical carcinoma is extremely rare, and unilateral tumors confined to one cervix are even more exceptional (Corbett et al., 1982, Gomez-Irizarry et al., 1996). Current evidence suggests that Müllerian anomalies do not increase inherent oncologic risk; rather, altered anatomy compromises screening and early detection.

Comprehensive evaluation of suspected Müllerian anomalies is therefore essential. While transvaginal ultrasound may raise suspicion of uterine malformation, pelvic MRI is considered the reference standard for precise delineation of uterine morphology and cervical anatomy, enabling appropriate classification according to ASRM and ESHRE/ESGE systems. In addition, systematic assessment for associated genitourinary tract anomalies is recommended due to their shared embryologic origin. In the present case, prior evaluation relied on ultrasound and hysteroscopy without MRI correlation, which likely contributed to incomplete recognition of cervical duplication and vaginal septum. No renal or urinary tract anomalies were identified.

The literature describing unilateral invasive cancer is limited. Early publications focused on precursor lesions or bilateral cancers. Corbett and Crompton (1982) documented the first invasive carcinoma restricted to a single cervix, supporting a role for localized HPV exposure when only one hemivagina is functionally accessed during intercourse (Corbett et al., 1982). The same mechanism is plausible in our patient.

Since then, a small but heterogeneous group of unilateral cases has been described, encompassing both didelphys and septate uteri with cervical duplication (Cordoba, 2017, Guo, 2011). Reports include advanced presentations (Gomez-Irizarry et al., 1996, Watanabe, 2012), mixed bilateral pathology (Lee, 2000), and early-stage lesions successfully treated with radical surgery (Kimball et al., 2006, Kaba, 2011). More contemporary studies illustrate the contribution of modern imaging and classification to individualized surgical planning (Valdespino, 2019, Gong et al., 2022). Importantly, several pre-2013 reports lack precise anatomic descriptions, raising the possibility of misclassified septate or bicornuate uteri previously labeled as didelphys (Grimbizis, 2013).

The comparative synthesis of these cases, summarized in Table 1, highlights two essential patterns: marked anatomic variability, with most cases involving didelphys or complete septate uteri with duplicated cervices; and consistency in tumor laterality, with disease typically confined to a single cervix despite structural duplication. These findings support the concept that HPV exposure and independent epithelial microenvironments, rather than the anomaly itself, drive carcinogenesis in duplicated cervices.

**Table 1:** Published cases of unilateral invasive cervical carcinoma in patients with cervical duplication.

**Reference**	**Age / Parity**	**Müllerian malformation**	**Cervical lesion pathology**	**Treatment**	**Follow-up**
**Corbett et al (1982)**	56, G0	Uterus didelphys, two vaginas and two cervices	Poorly differentiated squamous cell carcinoma, right cervix (IB)	Preoperative RT + RH + LND	NR
**Gomez-Irizarry et al (1996)**	44, G0	Uterus didelphys, two vaginas and two cervices	Moderately differentiated adenosquamous carcinoma, left cervix	RH + BSO + pelvic and *para*-aortic LND + omentectomy + CRT + CT	10 months (death)
**Lee et al (2000)**	45, G1P1	Uterus didelphys, two vaginas and two cervices	Invasive carcinoma, left cervix (IIA1)	LEEP + RT	4 years
**Kimball et al (2006)2**	39, G2P2	Septate uterus, two vaginas and two cervices	Squamous cell carcinoma, right cervix (IB1)	RH + pelvic and *para*-aortic LND	At least 4 months
**Guo et al** **(2011)2**	40, N/A	Septate uterus, two vaginas and two cervices	Invasive carcinoma, left cervix (IIA)	NR	NR
**Watanabe et al (2012)**	33, G2P2	Uterus didelphys, two vaginas and two cervices	Squamous cell carcinoma, right cervix (IVA)	CT (paclitaxel + carboplatin) + RT + anterior exenteration	NR
**Kaba et al (2012)**	49, G2P2	Uterus didelphys, two vaginas and two cervices	Well-differentiated endometrioid adenocarcinoma, right cervix (IB1)	RH + BSO + pelvic and *para*-aortic LND + omentectomy	15 months
**Cordoba et al (2017)**	37, G7P2	Uterus didelphys, two vaginas and two cervices	Adenocarcinoma, left cervix (IIIA)	CRT	At least 2 years
**Valdespino et al (2019)**	55, G4P4	Uterus didelphys, two vaginas and two cervices	Squamous cell carcinoma, right cervix (IB1)	RH (right side) + TH (left side) + pelvic and *para*-aortic LND	NR
**Gong et al (2022)**	40, G4P1	Septate uterus, two vaginas and two cervices	Squamous cell carcinoma, left cervix (IA1)	TH + salpingectomy	At least 6 months

Legend: **RH** = Radical hysterectomy, **TH** = Total abdominal hysterectomy, **BSO** = Bilateral salpingo-oophorectomy, **RT** = Radiotherapy, **CRT** = Chemoradiotherapy, **CT** = Chemotherapy, **LND** = Lymphadenectomy, **NR** = Not reported, **LEEP** = Loop Electrosurgical Excision Procedure.

These observations further underscore the importance of primary prevention. Prophylactic HPV vaccination remains a cornerstone in reducing the global incidence of cervical cancer. This consideration may be particularly relevant in women with Müllerian anomalies, in whom complex anatomy can compromise adequate cytologic screening and delay early detection. In such cases, vaccination assumes additional significance as a preventive strategy independent of screening limitations.

Management generally follows conventional principles used to guide therapeutic intervention in patients with cervical cancer without the presence of Mullerian duct anomaly. However, the rarity of the co-existence of cervical cancer and Mullerian duct anomalies combined with the wide range of clinical presentations places a premium on multi-specialty case management conferences, as was utilized in this case, to provide a consensus-based treatment recommendation. Therapeutic individualization is particularly important, if not an absolute requirement when anatomic variation is so diverse that the ability to blindly apply standardized radiation therapy, particularly brachytherapy or surgery to treat these conditions, would likely compromise outcomes. As a result, individualization of treatment is a point stressed in virtually every publication on this topic, including the present one. Particular attention should be paid to issues related to the presence and position of vaginal septae, surgical access and parametrial relationships, as they pertain to the use of brachytherapy as well as integrating these findings with the application of selection criteria used to determine suitability for surgical intervention. In the present case, radical hysterectomy with bilateral salpingo-oophorectomy, and lymphadenectomy was recommended by a muti-specialty panel as feasible and potentially curative. Additional chemoradiation was administered due to the identification nodal metastasis. The patient's favorable long-term outcome aligns with the limited published experience for unilateral cases treated with curative intent (Kimball et al., 2006, Valdespino, 2019). Recognition of Müllerian variants is critical to avoid diagnostic oversight and ensure appropriate cervical evaluation. This case underscores the importance of meticulous anatomic assessment in patients with duplicated cervices and cancer, as well as the utilization of multiple specialists to provide a consensus in order to guide appropriate evaluation and treatment.

## 4 Conclusion

Congenital Müllerian anomalies are uncommon, with septate uteri accounting for more than half of these malformations. This case illustrates a complete septate uterus with cervical duplication and a longitudinal uterovaginal septum. Given the scarcity of data and the classificatory challenges surrounding these anomalies, this report highlights the importance of accurate early identification, not only for reproductive implications but also for oncologic safety. Unrecognized cervical duplication may compromise cytologic screening and delay the diagnosis of cervical neoplasia.

Clinicians should remain vigilant to the diagnostic and management challenges imposed by these anatomic variants. Early recognition is essential to prevent screening failures and ensure appropriate treatment, underscoring the relevance of this case to clinical practice and the existing literature.

## Ethics statement

Written informed consent was obtained from the patient for publication of this case report.

## CRediT authorship contribution statement

**Camilla Bandeira Soares:** Writing – review & editing, Writing – original draft, Validation, Project administration, Methodology, Investigation, Formal analysis, Data curation, Conceptualization. **Nick M. Spirtos:** Writing – review & editing, Supervision, Conceptualization. **Joao Jabbur Stern:** Writing – review & editing, Visualization, Data curation. **Marcelle Almeida Souza:** Writing – review & editing, Investigation, Data curation. **Felipe Jacyntho Laterça:** Writing – review & editing, Investigation, Data curation. **Gustavo Guitmann:** Writing – review & editing, Validation, Supervision, Methodology, Investigation, Data curation, Conceptualization.

## Declaration of competing interest

The authors declare that they have no known competing financial interests or personal relationships that could have appeared to influence the work reported in this paper.
